# Management of Stromal Corneal Dystrophies; Review of the Literature with a Focus on Phototherapeutic Keratectomy and Keratoplasty

**DOI:** 10.3390/vision7010022

**Published:** 2023-03-13

**Authors:** Zahra Ashena, Magdalena Niestrata, Shokufeh Tavassoli

**Affiliations:** 1 Ophthalmology Department, Queen’s Hospital, Barking, Havering and Redbridge University NHS Hospitals Trust, Romford RM7 0AG, UK; 2 Moorfields Reading Centre and Clinical AI Hub, Moorfields Eye Hospital NHS Foundation Trust, London EC1V 2PD, UK; 3 NIHR Biomedical Research Centre, UCL Institute of Ophthalmology, London EC1V 2PD, UK; 4 Ophthalmology Department, Royal United Hospital, Bath BA1 3NG, UK

**Keywords:** stromal dystrophy, phototherapeutic keratectomy (PTK), penetrating keratoplasty (PK), deep anterior lamellar keratoplasty (DALK)

## Abstract

Corneal dystrophies are a group of non-inflammatory inherited disorders of the cornea. This review considers treatment options for epithelial-stromal and stromal corneal dystrophies: namely Reis–Bücklers, Thiel–Behnke, lattice, Avellino, granular, macular and Schnyder corneal dystrophies. Where there is visual reduction, treatment options may include either phototherapeutic keratectomy (PTK) or corneal transplantation. Due to the anterior location of the deposits in Reis-Bücklers and Thiel–Behnke dystrophies, PTK is considered the treatment of choice. For lattice, Avellino, granular and macular corneal dystrophies, PTK provides temporary visual improvement; however, with recurrences, repeat PTK or a corneal transplant would be needed. For Schnyder dystrophy, should treatment be required, PTK may be the preferred option due to the potential for recurrence of the disease in corneal transplantation. This review discusses the literature and evidence base for the treatment of corneal dystrophies in terms of visual outcomes and recurrence rate.

## 1. Introduction

Corneal dystrophies are a group of non-inflammatory inherited disorders of the cornea that are caused by the progressive accumulation of deposits within the layers of the cornea, secondary to the genetic mutations, which lead to the transcription of aberrant proteins, usually without systemic involvement. The disorders may affect the vision and may or may not be symmetrical [1]. Corneal dystrophies may have a simple autosomal dominant, autosomal recessive or X-linked recessive mode of inheritance. They present with variable-shaped corneal opacities in a clear or cloudy cornea, affecting visual acuity to different degrees. Diagnosis can be established on clinical grounds and may be enhanced with studies on surgically excised corneal tissue and, in some cases, with molecular genetic analyses [2].

The International Committee for the Classification of Corneal Dystrophies (IC3D), in its latest classification in 2015, classified corneal dystrophies into four groups: epithelial and subepithelial dystrophies, epithelial-stromal *TGFBI* dystrophies, stromal dystrophies, and endothelial dystrophies [3].

The treatment approach varies based on the type, symptoms and severity of each dystrophy. However, it is well understood that none of the treatments is permanent, and the disease recurs within a few months/years post-operatively. The literature lacks a review of stromal dystrophies focusing on the management plan. Considering the similar manifestations of epithelial-stromal and stromal dystrophies, in this review, we look at the treatment outcomes for seven epithelial-stromal and stromal corneal dystrophies, focusing on the role of phototherapeutic keratectomy (PTK) and keratoplasties in improving the symptoms and visual outcome as well as assessing the likelihood of a clinically significant disease recurrence with each treatment modality.

To achieve this, a literature review was conducted, and publications on the surgical management of Reis–Bücklers, Thiel–Behnke, Schnyder, lattice, Avellino, granular, and macular corneal dystrophies, with a focus on the outcome of PTK and keratoplasty, were collected on PubMed and Embase. The studies were categorised, and the outcome and prognosis of treatment were assessed for each group.

## 2. Reis–Bücklers Corneal Dystrophy

Reis–Bücklers corneal dystrophy (RBCD) is a bilateral and autosomal dominant disease caused by a mutation in keratoepithilin, i.e., *BIGH3*, also known as transforming human growth factor (*TGFBI*), on chromosome 5*q*. This presents early in childhood or teenage years with recurrent painful erosions [4]. In the more advanced form of the disease, corneal opacities form in a geographic pattern leading to a reduction in visual acuity. Visual acuity is also reduced due to acquired astigmatism secondary to corneal irregularity from scarring following multiple recurrent erosions and interestingly, near vision is relatively unaffected compared to distance vision [4].

The histopathological examination of corneas with RBCD reveals abnormal fibrocellular connective tissue replacing Bowman’s layer and growing anterior to it, forming intraepithelial projections, which, together with the surrounding scar tissue (Figure S1), create the biomicroscopic clinical appearances described above [5,6].

### 2.1. PTK

PTK has been used as an attempt to improve the vision in Reis–Bücklers corneal dystrophy in various studies. In this method, 193 nm ultraviolet light is used to ablate approximately 0.25 µm of corneal tissue [7]. PTK is less invasive than corneal transplantation, does not carry the risk of rejection, and can be repeated in cases of recurrence [8].

PTK improves the symptoms of recurrent erosions by debriding the irregularities and enhancing visual acuity by ablating the stromal opacity and removing corneal irregularities, thereby reducing astigmatism [7]. In cases of uniform anterior stromal opacities, they can be directly removed with the excimer laser, with the ablation continuing deeper into the stroma thereafter. In patients with significantly uneven epithelium, the ablation is performed following manual removal of the epithelium [7]. In cases of deep ablation, i.e., more than 50 µm of stroma, where a hyperopic shift is not desired, this can be counteracted by concomitant antihyperopic ablation [9,10].

An additional benefit of PTK is the ability to apply refractive correction (photorefractive keratectomy or PRK) in addition to removing the corneal deposits secondary to the corneal dystrophy [7].

Several authors have reported good visual and safe results from PTK in patients with Reis–Bücklers; however, the total numbers of treated cases were low [10,11,12,13,14,15]. Stark and colleagues reported visual improvement and recurrence at 2 years on two eyes with RBCD [10]. McDonnell and Seiler performed PTK on two patients with RBCD through the intact epithelium and achieved visual improvement and decreased symptoms of recurrent erosions [11].

Hahn et al. reported visual improvement at 9 months after PTK in one patient with RBCD whose eyes were both treated with no significant complications and no recurrence to date [12].

Lawless and colleagues treated nine eyes with RBCD with PTK, two of which previously had penetrating keratoplasty (PK) for the same indication. All patients achieved significant visual improvement and recurrent erosion symptoms resolved in all cases postoperatively. However, there were insufficient data on follow-up duration in this study [13].

El Aouni and colleagues treated 10 eyes with RBCD with PTK and achieved visual improvement for up to 1 year postoperatively [14]. Similarly, Kasetsuwan and colleagues reported four cases of successful PTK treatment in Reis–Bücklers dystrophy. The mean follow-up time was 9.9 months (range 6–18 months). Uncorrected visual acuity postoperatively improved in 88.2% of eyes, and ocular discomfort improved in 94.1% of eyes [15].

The above case series demonstrate examples of different stages of the disease being successfully treated with PTK. However, the overall numbers are small, and no guidelines or protocols exist to determine the patient’s suitability and prognosis based on how advanced the disease is, bearing in mind the required depth of ablation.

Pratik and colleagues described a case whereby a high ablation depth PTK was used to treat advanced RBCD where the patient had a thick opacity involving the epithelium, Bowman’s layer and anterior stroma that was 158 μm and 144 μm thick in the right and left eye, respectively. The authors performed a transepithelial ablation of the cumulative depth of 150 μm and 140 μm in the right and left eye, respectively. At 1 month, postoperatively, the patient’s corrected vision improved to 20/30 in both eyes, which was maintained at the 6-month follow-up [8]. This implies that it is feasible to successfully use PTK in advanced Reis–Bücklers’ corneal dystrophy.

Vinciguerra and colleagues showed a variation of a technique of sequential customised therapeutic keratectomy, which achieved a significant visual improvement in their cases from an average VA of 20/50 preoperatively to post-op average VA of 20/25 (*p* < 0.01) [16]. They showed a significant reduction in the coma aberration and no change in the spherical and trefoil as well as total higher-order aberrations. None of the patients required a corneal graft at approximately 9 months afterwards. Post-operative hyperopia of approximately 1D was common, in line with other authors.

The reported rate of sight-threatening complications post PTK in patients with RBCD is very low. Delayed epithelialisation, corneal scarring, residual corneal opacity, irregular astigmatism and monocular diplopia are uncommonly reported after PTK in eyes with RBCD [10,15].

The main disadvantage of PTK is post-op hyperopia up to 2D in around 80%, described by several authors from the beginning of PTK treatment until this date [4,8,11,14,15].

Oblique angle of the laser beam in the peripheral cornea (and hence decreased peripheral ablation), greater central corneal ablation in cases of deeper corneal pathology, and epithelial hyperplasia at the edge of the ablation zone may contribute to the significant hyperopia post-PTK [10,17].

According to the current literature, the disease recurrence after PTK is high [7,9,18]. Dinh and colleagues reported a recurrence rate of 37%, mostly occurring 12 months post-operatively in the 17 cases of RBCD treated with PTK, and an average recurrence time of 21.6 months post-PTK [9].

Some authors have postulated that mitomycin C (MMC) as an adjunct to PTK is effective in reducing the rate of recurrence and likelihood of dystrophy recurrence [19,20].

Ayres et al. treated two eyes with Reis–Bücklers corneal dystrophy with PTK augmented with MMC; one had recurrence at the margin of the treated area. Miller et al. reported a 73-year-old woman with visually significant Reis–Bücklers dystrophy who underwent PTK of her right eye with 0.02% adjunctive MMC for visual rehabilitation [19]. The left eye had previously undergone PTK on two occasions without MMC, and the dystrophy had recurred following each treatment. One year after the procedure, her MMC-treated cornea remained clear with no recurrence of Reis–Bücklers dystrophy. In Pratik and colleagues’ report, early asymptomatic recurrence was noted at 4 years after augmented PTK, which is a fairly long recurrence-free time [8].

Since these are only case reports or case series without any comparative studies, no definite conclusion about the added benefit of MMC can be drawn. In addition, it is worth highlighting that the early reports of PTK in Reis–Bücklers dystrophy may have included Thiel–Behnke cases, as no additional diagnostic tool, such as genetic testing, was available apart from the clinical assessment to confirm the diagnosis [12,21].

Another strategy to enhance visual outcomes and reduce recurrence rates was described by Vinciguerra and colleagues [16], who reported good outcomes from sequential customised therapeutic keratectomy in 14 eyes of eight patients with RBCD with a mean follow-up of approximately 5 years. The surgical technique involved peeling the epithelium and the subepithelial membrane in patients with RBCD with a surgical spatula and subsequent multiple customised sequential keratectomies with repeated excimer laser photoablations guided by multiple intraoperative topographies to target irregularities. The authors reported a fantastic disease recurrence rate of 14.28% (two eyes) after 5–6 years, which was managed by re-treatment with the same protocol.

Although PTK is currently the most commonly performed treatment for RBCD with a rapid visual improvement, as evident from the above, the literature evidence on the recommended treatment for RBCD is scarce and composed of case reports and case series only with no comparative trials, similar to the rest of the stromal/Bowman layer dystrophies.

### 2.2. Keratoplasty

The traditional approach to treating visually significant opacities secondary to anterior stromal dystrophies has been with keratoplasty, penetrating or anterior lamellar; however, the literature evidence on the recurrence and success rates, as well as visual outcomes, is scarce. Deep anterior lamellar keratoplasty (DALK) has the advantage of retaining the patient’s own endothelium and, therefore, reducing the risk of endothelial rejection and graft failure [22]. Nonetheless, the reported unaided visual outcomes after keratoplasty for RBCD are not comparable with those of PTK, due to irregular astigmatism [23]. The literature on the recurrence rate of RBCD after penetrating keratoplasty (PK) is very limited. Cadwell reported clinically significant recurrence in the graft 15 years post-op [24].

Another treatment modality that has been described is superficial keratectomy. Wood and colleagues reported treating three patients with this intervention, whereby fibrous corneal tissue was manually dissected. The advantages of the technique are its simplicity and benefit in improving the pain from recurrent erosions; however, the visual improvement post-operatively is modest [25].

Fogla and Knyazer described a variation of the lamellar keratoplasty technique resulting in good visual outcomes in patients with RBCD without the added endothelial rejection risk of a PK [26]. This group reported a microkeratome-assisted two-stage technique of superficial anterior lamellar keratoplasty (SALK) performed in four eyes of two patients with RBCD whereby a 9 mm, 140 μm thick corneal flap was created with a microkeratome in stage one. After 4 weeks, a 7.0 mm central trephination with a depth of 150 μm was performed within the pre-created flap using a Hessberg-Baron suction trephine. Subsequently, donor lamellar tissue was used to replace the host corneal defect in the flap without the need to suture. At an average 19 months postoperatively, there was no recurrence or graft rejection, and the best corrected visual acuity was 20/30 in both eyes. There was no statement on graft dehiscence or dislocation in their report [26].

In summary, due to the anterior location of the deposits, keratoplasty has rarely been used to treat RBCD. Hence, the literature on their outcome is very scarce.

Table S1 provides a summary of published papers on the outcome of PTK and keratoplasty in RBCD.

## 3. Thiel–Behnke Corneal Dystrophy

Thiel–Behnke corneal dystrophy (TBCD), otherwise known as honeycomb corneal dystrophy, is a bilateral autosomal dominant Bowman’s layer dystrophy caused by a heterozygous missense mutation of the *BIGH3* (also known as *TGFBI*) gene [27,28]. TBCD is characterised by honeycomb subepithelial corneal opacities macroscopically and ‘curly fibres’ on electron microscopy [29]. It tends to present at the age of 10–20 years with recurrent epithelial erosions [2,30].

Similarly to RBCD, the body of evidence regarding the treatment outcomes is small and comprises only case reports and case series with no comparative studies or randomised control trials.

The treatment goals in Thiel–Behnke corneal dystrophy are analogical to those of Reis–Bücklers dystrophy, with the therapeutical aims of reducing pain from recurrent corneal erosions and improving vision. The available treatment modalities include conservative treatment such as bandage soft contact lenses or surgical treatments, including stromal puncture, phototherapeutic keratectomy and lamellar or penetrating keratoplasty. The main aim is to improve vision by removing corneal opacities while delaying or eliminating the need for keratoplasty, which is associated with the risks of rejection, graft failure, cataract and glaucoma [31].

### 3.1. PTK

Similar to RBCD, PTK is a good treatment modality to remove corneal deposits and improve vision. As mentioned, it can induce hyperopic shift and less commonly cause delayed re-epithelialization, corneal haze and scarring, and irregular astigmatism secondary to a decentred ablation zone or uneven ablation due to pathological corneal opacities [32,33,34]. These side effects are more common with deeper ablations [34]. The other disadvantage of PTK compared to keratoplasty is that it does not allow for histopathological diagnosis of the excised tissue to confirm the diagnosis.

However, most authors have reported successfully treating TBCD with PTK.

Amm treated six eyes of five patients with TBCD, including four treatment-naive eyes and two eyes with recurrence of dystrophy in the graft with PTK. The diagnosis was made based on clinical appearances only, without genetic confirmation [35]. Significant visual acuity improvement was achieved in the follow-up period of 8 months to 4 years. Disease recurrence was reported in one eye, i.e., 16.7%, which had had a previous keratoplasty [35].

Sorour et al. treated eight eyes of four patients with a clinical diagnosis of Thiel–Behnke corneal dystrophy with PTK [34]. The group reported a 100% simple recurrence rate, but despite the recurrence, functional best-corrected central visual acuity was maintained, ranging from 20/25 to 20/80 in the long follow-up period of 9.7 years on average. This is because the recurrence started from the periphery and did not significantly involve the central cornea by the latest visit. Therefore, Sorour et al. advocate that the goal of PTK is not the complete removal of stromal opacities but rather removing enough abnormal tissue to allow for symptomatic relief from painful recurrent erosions and decreasing the visually significant opacity. In their cohort, only one eye needed repeat PTK.

Hieda and colleagues reported a case series involving 10 eyes of 10 patients with TBCD who were treated with PTK [36]. The authors showed a significant visual improvement of 0.54 LogMAR within an average follow-up period of 12 months. The disease recurrence rate was 50% (five out of 10 eyes) within that follow-up period, with a significant reduction of visual acuity by two lines, in four out of five patients with recurrence.

Hsiao et al. described a variation to standard PTK that aimed at reducing the post-PTK hyperopia by using combined wavefront-guided photorefractive keratectomy (PRK) combined with excimer laser PTK in one TBCD patient [33]. The treatment was successful and resulted in significant vision improvement in both eyes.

In summary, compared to keratoplasty, PTK is a relatively safe, simple and repeatable treatment [37] with a short recovery time (with the provision that no more than a third of the cornea is removed, i.e., up to 100 µm with the minimum remaining stromal thickness of 300–250 µm) [36]. Although the recurrence rate following treatment is high, it often is not visually significant for a while and is lower than the recurrence rate with RBCD [36].

### 3.2. Keratoplasty

The literature on keratoplasty in TBCD is scarce and involves case reports and case series with no comparative studies or randomised controlled trials.

Sorour et al. reported treating two eyes of one patient with TBCD with penetrating keratoplasty, compared to eight eyes treated with PTK in the same study [34]. The criteria for performing both surgeries were the same—reduced visual acuity despite maximum medical therapy—and the authors did not outline the reasoning of why each procedure was chosen in the particular patients. Additionally, no subgroup analysis was performed for eyes with PTK and PK.

Recurrence of Thiel–Behnke dystrophy is heterogeneous and often mirrors the pattern of the original dystrophy, affecting the periphery first and spreading to the central cornea, which could be due to the potential epithelial origin for the pathogenesis of TBCD recurrence [34].

### 3.3. FLK

Another technique that has been described to improve vision without post-operative hyperopia is femtosecond laser-assisted superficial lamellar keratectomy. Abusayf et al. reported successfully treating a case of TBCD with a femtosecond laser (FLK) [38]. The depth of the corneal opacity was determined using anterior segment optical coherence tomography (AS-OCT), and femtosecond laser-assisted superficial lamellar keratectomy (FSASLK) was performed by creating a free corneal cap of 9 mm in diameter using a femtosecond laser. The corneal cap thickness was selected at 120 μm. Visual improvement from CF to 20/100 was achieved with that technique in an eye with a very guarded prognosis and ocular comorbidities, including aphakia and previous retinal detachment surgery.

In summary, provided there is no significant central corneal thinning and the opacity is not deeper than 1/3 of the cornea, PTK is the treatment of choice and can be repeated if recurrence happens. If PTK is not feasible, FLK or keratoplasty could be tried. Nonetheless, there is no comparative study in hand to be able to provide a definite guideline on the management plan. Specific genetic makeup and mutation may help provide a more accurate prognosis. However, more studies are needed to confirm that.

Table S2 provides a summary of published papers on the outcome of PTK and keratoplasty in TBCD.

## 4. Lattice Corneal Dystrophy

Lattice corneal dystrophy (LCD) is an autosomal dominant corneal stromal dystrophy caused by a mutation in the *TGFBI* gene. The condition is characterised by amyloid deposits in the stroma, which stains orange-red with Congo red and green with periodic acid-Schiff (PAS), Masson trichrome and fluorochrome thioflavin T [39]. They result in progressive visual loss and recurrent corneal erosions, which are the most common presenting features.

Multiple subtypes of lattice dystrophy have been described depending on the specific genetic variation. Broadly speaking, it can be classified into Type I LCD (LCD1), also known as classic lattice corneal dystrophy or Biber–Haab–Dimmer dystrophy, and LCD type II, which is primarily a systemic disorder of amyloid deposition in different organs, including eye in the corneal stroma, and is more often referred to as familial amyloid polyneuropathy (FAP) type IV or Meretoja syndrome. The systemic characteristics include neuropathy, commonly facial nerve palsy, and significant skin laxity [40].

Another disease process often mistakenly included in the LCD family is granular corneal dystrophy type II (GCD type II), combined granular-lattice dystrophy or Avellino dystrophy, which will be discussed separately.

### 4.1. PTK

Several authors have reported favourable results of PTK in isolated patients with lattice dystrophy [9,18,21,41].

Hieda and colleagues reported treating 13 LCD patients with PTK using the ablation depth of 106.1 ± 9.8 μm and reported significant visual improvement within the mean follow-up of 44.8 ± 25.6 months [42]. They reported a clinically significant recurrence rate of 30.1% among these eyes. However, the recurrence was slow, with the median time from surgery to observed recurrence of 96 months.

Das et al. treated 12 eyes with lattice dystrophy by applying PTK following epithelial debridement and pannus removal [43]. They reported visual improvement in 62% by a mean of LogMAR 0.16 ± 0.15. They noticed a clinically significant recurrence in 17% of cases at the mean follow-up of 3.0 ± 2.7 years. One eye needed repeat PTK and one eye underwent keratoplasty for recurrence.

Dinh et al. treated seven eyes with lattice dystrophy, five of which had a previous PK, with PTK with only one case of recurrence (14%). However, the follow-up period was relatively short, at an average of 15 months [9].

Chiambaretta and colleagues treated 19 eyes with lattice dystrophy with PTK. They reported significant visual improvement and the disappearance of repeating ulcerations within the follow-up period of 36 months [44]. Likewise, Sauvegeot et al. reported treating 22 virgin eyes with LCD and 10 eyes with the previous PK for LCD, with PTK with good visual outcomes [45]. The mean follow-up was 5.2 ± 3.9 and 3.8 ± 2.4 years in the first and second subgroups. Visual acuity improved in all 22 eyes. They reported a recurrence rate of 41% after 5 years and 68% after 10 years in the virgin eyes. The mean time to recurrence was 6.2 years, and 59% of eyes needed a second PTK, which was followed by another recurrence after an average of 4.5 years. In the post-PK group, recurrence was seen in 10%, 30%, and 50% of eyes at 1 year, 5 years and 10 years post-op, respectively. The mean time to recurrence was 5.1 years. Two of those eyes with recurrence needed a second PTK [45].

### 4.2. Keratoplasty

Compared to the Bowman layer dystrophies, the indication for keratoplasty in LCD, owing to the deeper corneal involvement, is much higher. Mohamed et al. reviewed records of 72 eyes that underwent keratoplasty for lattice dystrophy with penetrating keratoplasty in 58 eyes and deep anterior lamellar keratoplasty in 14 eyes [46]. They found the visual outcome following DALK to be superior to that of PK, with a median BCVA of 0.14 and 0.4 LogMar at 3 years post-op after DALK and PK, respectively. As expected, the rate of graft rejection in DALK was lower than in PK, with 7% and 24%, respectively. Similarly, graft failure was less common in DALK (14%) compared with PK (24%).

Kawashima and colleagues compared the outcomes of DALK and PK in 60 eyes with Lattice dystrophy consisting of 31 DALKs and 29 PKs [47]. They found no significant difference in BCVA post-op between DALK and PK patients; nonetheless, for the purpose of that analysis, they reported the VA results for macular and lattice dystrophy patients together. Hence, it is impossible to infer whether there was a difference in the lattice dystrophy group. In addition, they reported a very small percentage of transient rise in intraocular pressure post-DALK, which did not lead to secondary glaucoma, whereas the rate of secondary glaucoma post-PK was 17%. There were no episodes of rejection reported in the DALK group, compared to a 20.6% rejection rate in the PK group. The endothelial cell loss rate was the same in both groups, and in terms of graft failure, there were no instances in the DALK group, while one case failed due to immunological rejection in the PK group.

Unal et al. performed big-bubble DALK on 74 eyes with corneal dystrophy, including 18 eyes with lattice dystrophy [48]. They noticed a high rate of disease recurrence (35.3%) in this group of patients, with a mean time to recurrence of 33.0 ± 7.7 months in the entire cohort. The recurrence rate of LCD with PK in Meyer’s study is comparable to the above study, as they noticed 33% recurrence in 21 eyes with LCD treated with PK [49]. In a longer follow-up, Meisler and Fine reported a higher recurrence rate of 48% based on 61 eyes that underwent PK for LCD during a follow-up period ranging from 3 to 26 years [50].

Spelsberg and colleagues (2004) conducted a comparative study of limbo-keratoplasty versus PK in patients with lattice dystrophy, in which 17 eyes with lattice dystrophy underwent limbo-keratoplasty and the recurrence rate was compared to the historical data from patients that had undergone PK (*n* = 16) [51]. There were two recurrences (11.8%) in limbo-keratoplasty eyes with lattice dystrophy, compared to six recurrences in PK eyes (37.5%). No instances of graft failure were reported in the limbo-keratoplasty group, and although the difference was not statistically significant, limbo-keratoplasty appeared to be associated with fewer recurrences. The authors speculated that this may be due to survival of transplanted limbal cells over several years, as confirmed by the genetic analysis. The authors did not report any significant complications of the procedure. With only one study to date, the evidence is insufficient for a clear change in practice; however, limbo-keratoplasty may be a consideration as a therapeutic choice pending more studies in the future.

An important consideration while analysing the recurrence rate with DALK is the surgical technique. The dissection technique varies from study to study, ranging from removing stromal tissue with viscodissection of Descemet’s membrane, big bubble technique, or manual dissection [47,48]. This can have a role in the recurrence rate post-keratoplasty.

Reinhart et al. in a literature review suggested that recurrence of the dystrophy post-DALK may be related to retained host stroma and would be less likely with Descemet membrane-baring techniques [52]. This suspicion of the stromal genesis of recurrence was also raised by Yao et al., who found, through histopathological and ultrasound examination, that incomplete removal of the recipient stroma during DALK could cause recurrence of lattice dystrophy [53]. A randomized trial would be needed to compare the outcome of DALK with different surgical techniques.

DALK has better transplant survival secondary to a lower risk of glaucoma, endothelial cell loss, and immunological rejection. However, theoretically, it can have a higher risk of recurrence owing to residual genetically defected host cells in the graft–host interface. More comparative prospective studies are required to find the answer.

### 4.3. FLK

A variation of PTK with recent technological advancements is femtosecond laser-assisted lamellar keratectomy (FLK), which enables the selective removal of layers of tissue, leaving a smooth stromal bed of reasonable optical quality [32]. In this technique, the excised flap can have a histopathological examination to confirm the diagnosis. Only a few cases are described to date in the literature. Steger and colleagues described FLK in one patient with lattice dystrophy. Lee et al. successfully treated three eyes of two patients with lattice dystrophy with FLK and additional MMC to smoothen the surface [54]. The vision in all three eyes reached up to 20/25 post-op. Recurrent corneal erosion (RCE) did not occur during follow-up in any of the eyes. In one patient, irregular astigmatism (secondary to the previous PTK) was successfully corrected with FLK. After the procedure, BCVA improved to 20/25 up to 6-year follow-up, and irregular astigmatism improved from 4.2D to 1.9D.

In summary, PTK is a beneficial treatment offering good visual outcomes and delaying the need for keratoplasty. The recurrence rate of lattice dystrophy after any treatment is high. However, the time to recurrence is relatively slow, allowing several re-treatments.

Therefore, to maximise the visual acuity over a lifetime, it is recommended to start with PTK and retreat with PTK before proceeding to keratoplasty when possible. FLK appears to be a beneficial option that also allows for the correction of irregular astigmatism at the same time. However, evidence to date is still quite limited.

When moving to keratoplasty, DALK is preferred to PK for better graft survival; however, the recurrence rate is not fully understood yet.

As evident from the literature, there is substantial variability in patients’ outcomes in terms of post-op visual acuity and recurrence rates among patients treated with the same treatment modalities. Ellies and colleagues suggest that this variability can be explained by specific genetic mutations responsible for the disease within each dystrophy group. They showed a high correlation between the clinical outcome and the genetic mutation [55]. The presence of specific mutations was characterised by an early need for treatment and early disease recurrence. At the same time, some other mutations were associated with slower progression and slower recurrence post-treatment. The authors suggested that defining the molecular defect responsible for the disease is essential in helping to predict the adequate treatment, which should, therefore, be individualised for each patient.

Subsequently, the same group reported treating a large cohort of 42 eyes of 29 patients with *BIGH3*-linked corneal dystrophies and previous penetrating keratoplasty with PTK. The authors showed that the magnitude of visual acuity improvement depended on the specific mutation [56].

Table S3 summarises published papers on the outcome of PTK and keratoplasty in LCD.

## 5. Granular Dystrophy

Granular corneal dystrophy (GCD) type 1 is an autosomal dominant disease with hyaline deposition in the corneal stroma [57]. In fact, it represents one of the most common hereditary corneal dystrophies with an autosomal dominant trait [58]. The disease manifests with deposition of sharply demarcated greyish-white lesions, which initially appear in the central basal epithelium and anterior stroma and, with time, become denser and progress more posteriorly and peripherally; however, the peripheral cornea usually remains free of deposits (Figure S2) [58,59]. The deposits appear in the first or second decade of life; however, visual impairment usually starts around the fifth decade of life [58,59]. PTK, corneal transplantation, and other treatment modalities have been used to restore vision in this dystrophy.

### 5.1. PTK

PTK has been shown to produce a good visual outcome in eyes with GCD, based on various case reports and series [9,41,60,61]. Nassaralla et al. reported a good outcome of PTK, using the ablation depth of 110 μm, in two patients (in their 40s and 50s) with GCD. They noticed a hyperopic shift and mild haze in their patients but no recurrence after 36 and 48 months [41]. This is one of the early reports of PTK; hence, no MMC or PRK was applied to prevent the haze and hyperopia. Another old report of PTK in GCD dates back to 1998, with a superficial ablation depth of 35–70 μm and a medium follow-up duration of 32 months in five eyes. The vision did not improve in two eyes, and another eye had a significant recurrence after 52 months of follow-up [18]. Another case series reported 13 eyes with GCD, two of which had previous PK [9]. The ablation depth was decided individually based on the extent of stromal involvement; however, no data were provided on the average ablation depth. There was a 23% rate of significant recurrence after 40 months post-op. They advocated PTK as a safe treatment option in virgin eyes, as well as transplanted eyes, to restore vision before proceeding with more invasive treatments such as DALK or PK.

The visual outcome of PTK was evaluated in a large-scale study with 50 eyes with granular dystrophy and an ablation depth of 15–110 μm. The vision improved in 79% of patients. They were followed up for an average of 3 ± 2.7 years, with 20% recurrence rate. However, no definition of recurrence was provided, so it is not certain whether the recurrence was simple or significant [43].

Chen et al. studied the recurrence of corneal dystrophies in 44 eyes, including 15 eyes with GCD, that underwent PTK with an ablation depth of 30–100 μm [62]. Similarly to the previous studies, significant recurrence was defined as a loss of two or more lines on a Snellen chart or more than three episodes of pain, photophobia, tearing, or redness related to the original disease. They used RTvue OCT to study the depth and breadth of stromal involvement and confocal microscopy to assess the cellular and structural alterations in recurrence after the PTK. They reported an average of 23.7 ± 11.2 months until a significant recurrence in their cohort of eyes with GCD. Structural changes included disorganized stromal fibres and disordered arrangement of nerve fibres as well as decreased keratocyte density and endothelial cell density. Five eyes had repeat PTK, and six eyes proceeded with PK. The factors contributing to disease recurrence in their entire cohort were young age at the onset of the disease (less than 30 years), residual deposits after the first PTK procedure, laser ablation depth less than 50 μm, or a small ablation zone (less than 5 mm). They noticed the recurrence of the disease even in the presence of complete ablation of deposits. Their impression was that recurrence derived from the ablation margins and progressed aggressively to the centre.

Lewis et al. reported the outcome of PTK in 12 eyes with GCD [59]. The PTK ablation depth ranged between 68–256 μm with an average of 156 μm. There was a significant improvement in visual acuity 1.8 months post-operatively, with a median time to significant recurrence of 2.7 years.

PTK has also proved safe in eyes with a corneal graft. One study specifically assessed the outcome of PTK in eyes with a previous PK and various *BIGH3*-linked corneal dystrophies, including 14 with GCD, in which the disease had recurred in the graft [56]. They applied a genotypic approach and assessed the mutations in their patients using genetic tests. BCVA significantly improved in all groups; however, they noticed that the magnitude of visual improvement varied among patients with different mutations. PTK ablation depth of 80–90 μm was used and was not combined with a PRK to correct the induced hyperopia. There was one episode of graft rejection, which was well-controlled. The patients were followed up for 3.77 ± 1.23 years, with only one episode of recurrence after an average of 5.73 years, which is a good outcome. Maclean et al. reported a similar study of the outcome of PTK in three eyes with previous PK, in which the disease had recurred in the graft [63]. After removing the epithelium, PTK was performed with an ablation depth of 19.8, 22, and 28 μm. Due to the superficial ablation, there was no induced hypermetropia, hence allowing a fast visual recovery and a significant improvement in visual acuity in all patients without any harm to the grafts, i.e., graft rejection. The eyes were noticed to develop a mild, visually non-significant and transient haze post-operatively. No recurrence was noticed in a short-term follow-up of 7 months; however, the condition recurred in two eyes 2 years later, for which repeat PTK was performed. Rathi et al. assessed the safety of repeat PTK on 10 eyes with previous PK [64]. They used an ablation depth of 43.66 ± 19.57, 75 ± 43.30 and 39 ± 19.79 μm in the first, second and third PTK, respectively, without any application of MMC.

BCVA significantly improved after each procedure. There was one episode of graft rejection, which was successfully managed with medications.

Based on the above findings, PTK is a safe and repeatable treatment modality in eyes with a corneal transplant and obviates the need for repeat grafting. The safety profile of PTK in transplanted eyes with recurrence of granular and macular dystrophy was examined by Reddy et al. too [65]. They compared the visual gain and significant recurrence of the disease in two groups of patients with and without a previous corneal transplant. Both groups achieved equally good visual outcomes with an ablation depth of 88 ± 21 μm in virgin eyes and 71 ± 24 in transplanted eyes. Additionally, no corneal rejection was noticed, and despite having a longer follow-up in the transplanted eyes, the recurrence rate was the same in both groups.

Szentmary et al. examined the safety of PTK from another aspect [66]. They looked at the impact of PTK on eyes with granular and macular dystrophy that subsequently underwent PK later. The eyes had a PTK with an ablation depth of 41 ± 29 μm. They had eight eyes in the study group, all of which received a PK 3.7 ± 2.3 years post-PTK, and 13 eyes in the control group. During the postoperative follow-up, no eye developed immunological rejection. Moreover, there was no statistically significant difference between the BCVA and spherical equivalence between the groups. Therefore, PTK has no negative impact on the subsequent PK in eyes with granular and macular dystrophy.

PTK is reported to be associated with corneal haze and hypermetropia, in particular when a high ablation depth is applied to remove the opacities. Most studies showed that the haze decreased with treatment and did not affect the vision significantly. Few studies have used MMC in PTK for granular dystrophy [19,67,68]. The safety of applying MMC was assessed in Yuksel et al.’s study, where 18 eyes, including nine eyes with MCD and nine eyes with GCD, underwent PTK with an ablation depth of 117.8 ± 24.4 µm and 83.5 ± 45.7 µm, respectively, followed by 30 s of exposure to 0.02% MMC [69]. MMC did not delay the epithelial healing, and both groups showed a significant visual improvement. The significant recurrence rate was 11% in both groups after 22 and 20 months of follow-up.

MMC can reduce haze [68]. There are some claims about the role of MMC in delaying the recurrence, as it can suppress the proliferation of stromal keratocytes and block subepithelial fibrosis of the cornea [19,54,68]. However, the number of patients and the duration of follow-up in such studies are very limited. Hence no solid evidence is available, at least concerning the recurrence of granular dystrophy.

Regarding hyperopia, combining PTK with PRK has been shown to be effective in preventing this refractive error in eyes with high ablation depth [68].

PTK is demonstrated to be repeatable if the stromal thickness allows. That being said, another study examined corneal biomechanics after PTK with an ablation depth of 45–50 μm in eyes with GCD [70]. Surprisingly, corneal hysteresis and corneal resistance factor significantly reduced after PTK. Moreover, these indices were significantly correlated with the central corneal thickness before and after PTK, suggesting that the preoperative and postoperative corneal thickness play a role in corneal biomechanical characteristics. This suggests that eyes with repeat PTK are at potential risk of ectasia. However, no long-term follow-up is in hand to detect the rate of ectasia. Moreover, it is frequently observed that the disease recurs sooner than ectasia develops, as it is a slow process. In fact, due to the short-term visual benefits of PTK, the patients will undergo keratoplasty before the manifestation of ectasia.

### 5.2. Keratoplasty

Similarly to other stromal dystrophies, keratoplasty is the long-term management plan for granular dystrophy. Frising et al. described an unusual case of granular dystrophy in which the disease manifested at the age of 5 years and proceeded to a corneal transplant a year later, something uncommon with granular dystrophy [58]. Furthermore, each time the patient received a transplant, it only remained clear for about 5 years, and a repeat transplant was indicated. Histological examination of the corneal button confirmed the diagnosis; however, molecular genetic analysis of the patient and his family members revealed a new genetic polymorphism. This study indicates different genetic varieties of each dystrophy, which may cause different behaviour in terms of the age of onset, the severity of the disease and the tendency to recur. Some suggest that GCD has an epithelial origin and may recur anteriorly [31,58]. This hypothesis has not been confirmed, though, as contradictory papers suggest otherwise, i.e., the role of residual keratocytes and the type of mutation have a role in the pattern of recurrence [57,71,72]. Pantanelli et al. reported a case of GCD with a recurrence of the disease 3 years following an 8 mm DALK. Anterior segment OCT demonstrated pre-Descemet hyaline deposits. PK was offered, and the corneal button histology confirmed that recurrent hyaline deposits were confined to the residual host stroma just anterior to the Descemet membrane, following Melles manual dissection; hence, residual keratocytes are still a source of recurrence [57]. Based on this observation, it may be inferred that Descemet-baring techniques are superior in removal of the residual host stromal cells. The authors suggest that PK may be superior to DALK, with less recurrence.

Kodavoor et al. achieved a good visual outcome in 10 eyes with GCD that underwent 7.75–8.25 mm DALK. All patients had statistically significant vision gain and no recurrence at 1 year post-op. Their case series also included 16 eyes with macular dystrophy, which showed results similar to those of GCD regarding post-op BCVA and recurrence rate. They used As-OCT to visualise the extent of posterior stromal involvement and only included cases with no Descemet or endothelial involvement. They advocate DALK in such cases. However, their follow-up period was short, and longer follow-up studies would be indicated to confirm that.

Lyons et al. compared the efficacy of PK and DALK in 20 and 11 eyes with GCD, respectively [31]. The patients were followed up for an average of 91 months. Visual acuity after both procedures was not significantly different. Recurrence of the disease was noticed in all grafts between 13 and 73 months postoperatively, with the recurrence-free interval being no longer with PK, compared to DALK. The average time to repeat keratoplasty was 14 years. Although there was no discrete definition of a simple and significant recurrence, it can be inferred that a significant recurrence occurred at an average of 14 years. They found recurrence of the disease starting from the anterior stroma in a vortex pattern, hence suggesting the epithelial origin of this dystrophy.

Salouti et al. assessed the outcome of DALK, using the Melles dissection technique, in seven eyes with granular dystrophy [73]. They followed up with their patients for 38.4 ± 18.6 months and noticed a high recurrence rate of 43% at the average period of 38.4 ± 18.6 months. They concluded that the Melles dissection technique did not successfully remove all genetically defective host cells, hence a high recurrence rate. However, as the disease can potentially extend to the Descemet membrane, even lamellar dissection with a big bubble technique can be associated with a recurrence from the graft–host interface as of Oke et al. [71]. They noticed an early disease recurrence from the graft–host interface in two eyes with type 1 big bubble DALK and no recurrence up to 4 years after DALK with type-2 big bubble. They concluded that perhaps the removal of the host pre-Descemet layer potentially avoids early deep central recurrence of GCD. However, type-2 bubble is associated with a high risk of perforation. Based on these observations, they suggested a PK in deep stromal involvement of the GCD. Another study used the big bubble technique to bare the Descemet membrane in a cohort of eyes with stromal dystrophy, including one with granular and five with macular dystrophy, and reported a successful and complete baring of Descemet with no report of any recurrence [74]. However, they followed up their patients for 6 months only. Additionally, none of these studies provided any information on the posterior extent of the stromal dystrophies and whether patients with involvement of the Descemet membrane were excluded from DALK.

Lewis et al. reported the outcome of PTK and keratoplasty in a series of eyes with GCD. Twelve eyes underwent PTK, nine had anterior lamellar keratoplasty (ALK), six had DALK, and 23 eyes had PK [59]. They compared the mean time between the procedure and the significant recurrence of GCD in these patients. ALK was performed with either a microkeratome or femtosecond laser. For DALK, some eyes had a big bubble technique, and some had manual Melles dissection. DALK and PK graft size were 7.75–9 mm, and ALK graft thickness ranged between 120–250 μm with an average of 165 μm. The PTK ablation depth ranged between 68–256 μm with an average of 156 μm. The mean duration of follow-up was 11.7 years. Although there was a significant improvement in BCVA following the intervention, the median time to achieve BCVA was significantly longer in the PK group (5.3 months) and DALK group (8.4 months) compared with the PTK group (1.8 months). The median time to significant recurrence after PK was 13.7 years, ALK 3.7 years, DALK 3.2 years, and PTK 2.7 years. Recurrence was noticed centrally and in both the anterior and posterior stroma (graft–host junction). This was a good study with a high number of procedures and long-term follow-ups. They showed a long recurrence-free interval in their PK group, compared to the DALK/ALK and PTK groups. As it was a retrospective study, no criteria to select DALK over PK or vice versa were provided, and the posterior extent of the lesions in the DALK group was unclear.

In summary, various studies report various recurrence rates with keratoplasty. This may be due to the genetic heterogeneity of the disease, not differentiating between a simple and clinically significant recurrence, employing different techniques of lamellar dissection, and not excluding cases with posterior corneal involvement from DALK.

### 5.3. Other Treatment Modalities

Alcohol epitheliectomy with mechanical debridement of the granular deposits was suggested as an alternative to PTK, where PTK is not available [75]. In this report, both eyes of a child with superficial granular dystrophy were treated using PTK in one eye and the abovementioned method in the fellow eye. Both eyes gained good and equal visual acuity post-op, with hyperopia being a bigger concern in the PTK eye.

Chen et al. conducted a prospective study on the outcome of anterior lamellar keratoplasty (ALK) in eyes with previous PTK and recurrence of GCD. In their study, a microkeratome was used to cut the donor and recipient cornea and provide a partial thickness lamella [76]. They used 4–8 sutures to fix the graft on the recipient’s cornea. The patients were followed up for 18.9 ± 4.1 months. Visual acuity improved in all cases, and no recurrence was noticed during the short-term follow-up period.

Femtosecond laser-assisted lamellar keratectomy (FLK) is an alternative to PTK to remove the superficial stromal deposits with a femtosecond laser instead of a microkeratome [32]. With femtosecond laser, it is possible to treat a much larger corneal zone (9.5 mm) compared with PTK (usually 6.0–6.5 mm), thus avoiding transition zones that could potentially affect postoperative vision. In addition, the femtosecond laser produces planar rather than meniscus-shaped corneal flaps as in PTK, hence less likelihood of post-op hyperopic shift [77]. Steger et al. assessed the outcome of FLK on eight eyes, including four RBCD, one LCD, two MCD and one GCD, after 2 years of follow-up. The free cap thickness was set at 140 μm in these patients. The vision improved significantly in all patients with no induced hyperopia; however, the RBCD group experienced a much better best spectacle-corrected visual acuity (BSCVA). The patient with GCD showed a clinically significant recurrence 1.5 years post-op, while the rest of the eyes did not show any evidence of recurrence during the follow-up. Although FLK can be considered for the treatment of patients with anterior stromal dystrophies, it cannot be performed in eyes with deep corneal pathology, in which removal of greater than 20% to 30% of the corneal thickness would be required to clear the bulk of the opacity. Furthermore, due to the short follow-up duration, this study could not assess the true risk of ectasia.

It was noticed that following DALK, the recurrence may be subepithelial or from the posterior graft–host interface or the peripheral graft–host junction. The subepithelial recurrence of GCD prompted the theory of the epithelial origin of granular dystrophy. Therefore, limbo-keratoplasty, as opposed to PK, was advocated to ensure the epithelial layer of the graft originated from the donor rather than the host’s stem cells. Lang et al. reported two patients with GCD who underwent PK in one eye and limbo-keratoplasty in the other eye. In the first case, the eye with PK showed a significant recurrence 17 years post-op, while most of the fellow cornea remained clear 12 years post-op. In the second case, the eye with PK showed a recurrence at 18 months, while the eye with limbo-keratoplasty was still clear 5 years post-op [78]. Spelsberg et al. compared the outcome of limbo-keratoplasty in 16 GCD and 17 LCD cases with PK alone in 20 GCD and 16 LCD cases [51]. The patients were followed up for an average of 47 months. Although limbo-keratoplasty tended to be associated with fewer recurrences of granular and lattice dystrophies, the difference was not statistically significant. This was attributed to the probable disappearance of the transplanted limbal stem cells over time. Further studies are required to shed light on the significance of limbo-keratoplasty in reducing the recurrence of granular and lattice dystrophies.

FLK outcome was evaluated on four eyes in patients with GCD and previous PK followed by PTK to address a recurrence, in whom the dystrophy recurred again [79]. In this study, a 125–150 μm lamella with a diameter of 6.9–7 mm was inserted into the recipient’s cornea. Donor and recipient cuts were both performed with a femtosecond laser, and no corneal sutures were applied to fixate the graft; only a bandage contact lens was used. The eyes were followed up for 10–35 months. Vision improved by more than two lines in all eyes and remained the same until the last visit, with no evidence of visually significant recurrence. Additionally, there was no record of graft rejection or dehiscence.

In conclusion, to remove lesions that are confined to the anterior stroma, PTK may be offered and repeated after recurrence. FLK and automated laser keratectomy are alternatives to PTK in such cases. ALK and DALK are good choices where the stromal involvement is not too deep, and PK may be offered to restore the vision in deep stromal or pre-Descemet involvement.

Tables S4 and S5 summarise published papers on the outcome of PTK and keratoplasty in GCD.

## 6. Avellino Corneal Dystrophy

Granular corneal dystrophy (GCD) type 2, also known as Avellino corneal dystrophy, is a stromal corneal dystrophy that shares the clinical and histologic features of both granular and lattice dystrophy [80]. It was first described by Folberg et al. in 1988 [81]. It is an autosomal dominant condition inherited with very high penetrance. Characteristically, both hyaline granular deposits and amyloid lattice lesions occur, with later visual impairment being the result of an anterior stromal haze between the deposits [82]. The name Avellino corneal dystrophy originated from a report of two Italian-American families, the origins of which were traced to Avellino, Italy [82]. Subsequently, there have been many worldwide reports of the condition [83,84,85]. *R124H* mutation in the transforming growth factor beta-induced gene, *TGFB1*, or keratoepithelin gene in human chromosome 5 (5q31), occurs in patients with Avellino cornea dystrophy [86]. The diagnosis can be confirmed by using genetic analysis to demonstrate the replacement of histidine by arginine at codon 124 (*R124H*) of *TGFBI* [87]. Severity may vary between homozygous and heterozygous patients, with homozygous patients having corneal deposits at an earlier age and heterozygous patients showing a slower progression, with visual impairment developing only in later years [80]. There have been reports of progression of the condition following laser refractive surgery [85,88]. The majority of patients with Avellino dystrophy maintain their vision for many years. It may result in significant visual impairment only late in life. If necessary, these patients undergo keratoplasty or PTK, the latter of which may be a less invasive option. However, recurrence of the condition may occur with both treatment options.

### 6.1. PTK

PTK treatment, using an excimer laser system, has been described for use in Avellino dystrophy [89,90,91]. The PTK laser beam used in one study covered a 6 mm diameter circle centred on the pupil [89]. The depth of the ablation was guided by the extent of the opacity and the depth of the affected stroma. The technique involved trans-epithelial ablation, followed by stromal ablation, until corneal deposits disappeared within a permissible range through the microscope (mean ablation depth used for homozygous cases was 90.0 ± 20.0 μm, range, 60.0–100.0 μm) [89].

Recurrence of Avellino dystrophy can occur following PTK [89,90]. Stress-induced cellular changes and subsequent keratocyte stimulation may represent the stimulus leading to the deposition of abnormal material in Avellino dystrophy after excimer laser treatment [92]. The recurrence interval following PTK treatment has been reported as 7 to 9 months [90]. There are reports of a difference in recurrence interval following PTK, between homozygous and heterozygous cases of the condition [89]. Interestingly, one study found the mean recurrence-free interval to be significantly shorter for homozygous cases of Avellino dystrophy, compared to that of heterozygous cases (9.5 ± 3.1 months vs. 38.4 ± 6.2 months, *p* < 0.001) [89]. Furthermore, in this study, the clinical appearance of recurrent opacities differed between the homozygous and heterozygous mutation groups; those in homozygous patients started more diffusely from the peripheral cornea with a rapid centripetal spread, compared to a slower recurrence of lesions in the superficial central cornea with a small, discrete, and sharp demarcation in the heterozygous group of patients [89].

Moon et al. reported corneal deposits to reappear within 18 months after the primary PTK and within 3 months after the second or third PTK, with more severe recurrences with each subsequent treatment [91]. An explanation for this may be that corneal trauma elevates levels of *TGF*-beta, an inducer of the *big-h3* gene [91,93]. There were initial reports that PTK with 0.02% MMC could help prevent or delay the recurrence of heterozygous Avellino dystrophy exacerbated after LASIK [94]. However, a further study by the same group reported a case of recurrence at 2 months after PTK with 0.02% MMC in Avellino dystrophy [91]. Thus, the benefit of MMC with PTK to reduce the risk of recurrence in patients with Avellino dystrophy remains unclear and may not be of any additional benefit.

### 6.2. Keratoplasty

Surgical treatment with cornea transplantation is an option for improving vision in severe Avellino dystrophy. The cornea is generally clear immediately after surgery; however, recurrence in the form of deposits can start to appear in the corneal graft within 12–24 months [91]. Recurrence in the corneal graft was reported to occur along the suture tracts and incision lines in a case of PK for Avellino dystrophy. In cases of DALK, recurrence has also been reported to occur at the graft–host interface [91].

In a retrospective study of four eyes of four patients (mean age 52.5 ± 3.32 years), from a Korean population who underwent big-bubble DALK for Avellino dystrophy following multiple failed PTK treatments, there was one case of recurrence occurring at the peripheral graft–host interface at 13 months [95]. All cases had a post-operative BCVA of 20/25 or better, with a mean follow-up period of 17.5 ± 3.11 months [95].

No recurrence was reported to occur with IntraLase femtosecond assisted lamellar keratoplasty in a case of Avellino dystrophy in a 6-year-old male patient with a 19-month follow-up period [92]. Post-operative BCVA was 20/40. The authors suggest that the photodisruption effect with this longer wavelength laser and the smooth uniform cut, along with a more-smooth interface, may account for the reduced risk of corneal trauma and subsequently reduced risk of recurrence with femtosecond assisted lamellar keratoplasty. However, a longer duration of follow-up is required.

In summary, recurrence remains the greatest challenge in treating Avellino dystrophy. PTK laser treatment may be an initial option for cases with reduced visual acuity; however, with each subsequent treatment, there may be a greater risk of recurrence. Homozygous cases are likely to have a more severe and earlier recurrence. Cornea transplantation in the form of big-bubble DALK or femtosecond-assisted lamellar keratoplasty may be a suitable treatment option for visual improvement in cases with severe recurrence following PTK treatment.

Another interesting modality for restoring vision in Avellino stromal dystrophy is corneal electrolysis. This modality is helpful in removing the subepithelial deposits or those in the graft–host interface. In a case series by Mashima et al., four eyes were treated using a digital electrolyser with a flat electrode [96]. The lamella was lifted, and the deposits were removed using a flat electrode on both graft and host face, followed by repositioning the graft and fixing that with sutures. Visual acuity improved in all cases, and no graft rejection or significant recurrence was noticed after 6–36 months of follow-up.

## 7. Macular Dystrophy

Macular corneal dystrophy (MCD) is an autosomal recessive disorder, which is the least common dystrophy worldwide, except for South Asia, Iceland and Saudi Arabia, where it is commonly reported [97,98] and is attributed to consanguineous marriage [98]. A locus on chromosome 16 was found to be associated with MCD, where mutations in the carbohydrate sulfotransferase gene (*CHST6*) prevent normal sulfation of corneal keratan. Consequently, the deposition of abnormal proteoglycans in the stroma leads to decreased corneal transparency and decreased vision [99,100]. The onset of the disease is towards the end of the first decade of life, hence demanding treatment at a younger age compared to the other dystrophies [61]. It starts with an anterior fleck-like stromal haze in the centre of the cornea. Unlike granular corneal dystrophy, these flecks involve the limbus and deep stroma down to the Descemet membrane [99,101], where involvement of the Descemet membrane can cause the appearance of endothelial guttata [97]. Recurrent corneal erosion and blurred vision are the main manifestations of the disease [99]. Case reports, retrospective observations and prospective studies have discussed the safety and efficacy of PTK, keratoplasty and FLK in this type of stromal dystrophy.

### 7.1. PTK

A few sporadic reports, as well as case series and clinical trials, have been published on the safety profile and efficacy of PTK in macular dystrophy. Various studies have used different ablation depths based on the severity and extent of corneal stromal deposits and their local protocols. Anterior segment optical coherence tomography (AS-OCT) has been the main diagnostic tool to assess the posterior extent of dystrophy. However, some studies suggested that ultrasound biomicroscope (UBM) proved superior in detecting the posterior stromal involvement in MCD [101].

PTK tends to induce hyperopia as it flattens the central cornea; therefore, deeper ablations are associated with higher hypermetropia [102]. However, that can be combined with photorefractive keratectomy (PRK) to correct the hypermetropia [68]. Therefore, excellent short-term visual outcomes of PTK are reported in patients with MCD.

Nevertheless, the recurrence is almost definite after a few months to years. Significant recurrence, which is defined and widely accepted as a drop of two or more lines on a Snellen chart or showing more than three episodes of pain, photophobia, tearing, or redness related to the original disease [32,62], is reported at 18 months [102,103] and 26 and 40 months [61] post-procedure in patients with MCD after undergoing PTK.

Gassel et al. reported on a 32-year-old male who had a severe recurrence of the disease 18 months post-PTK [103].

Similarly, Shields et al. reported a case of a 16-year-old male with MCD, who underwent PTK with 35 μm stromal ablation after the removal of the epithelium, which was augmented by using MMC. Visual acuity improved to 6/9, but the disease recurred significantly 18 months later, so the patient was offered penetrating keratoplasty [102]. Kemer et al. published the outcome of augmented PTK in 14 eyes with MCD, two of which experienced significant recurrence 26 and 40 months later [61].

Wagoner et al. reported on a young patient who had a PTK in one eye, followed by PK in the other eye, with no recurrence of the disease in 2 years [97]. Studies with longer follow-ups, however, indicate that recurrence is inevitable with PTK. Hafner et al. reported 90% recurrence of macular dystrophy in 10 eyes that underwent PTK with an ablation depth of 20 to 100 μm and were followed up for 4.5 ± 3.1 years [104]. Another study evaluated the outcome of PTK in eyes with corneal dystrophies, including five with macular corneal dystrophy and nine with GCD, of which some had a previous history of penetrating keratoplasty [65]. They used the average ablation depth of 88 ± 21 μm in the virgin eyes and 71 ± 24 μm in transplanted eyes. PTK appeared to be a safe treatment in both groups with no complications, such as graft rejection, in transplanted corneas. The time to significant recurrence was 57 months in the MCD group compared to 38–43 months in the GDC group.

Chen et al. published the outcome of PTK in a group of patients with anterior corneal pathology, including four eyes with MCD [62]. An ablation depth of 30–100 μm was applied, and patients were followed up for an average of 95 months, with no specific information on the ablation depth and follow-up duration in MCD patients. They reported a clinically significant recurrence of MCD after an average of 13.5 ± 5.9 months, with recurrence appearing mainly within the ablation zone and gradually progressing to the deep stroma.

PTK appeared to be a safe and effective tool in treating recurrent disease in eyes with previous PK [65], as it helped postpone a repeat graft. In addition, it worked well in removing the anterior corneal stromal pathologies, including lesions secondary to stromal dystrophies, in children aged 6–8 years [105]. Moreover, it proved safe to be repeated in case of recurrence [37]. It showed no negative impact on the survival of the subsequent PK in eight patients with macular and granular corneal dystrophy [66]. Considering the excellent visual outcome and only short-term benefit of PTK in this very young cohort of patients, who have many years ahead of them, most studies advocate this treatment only to “buy time” and delay keratoplasty.

### 7.2. Keratoplasty

As mentioned earlier, MCD manifests earlier in life, compared to the other stromal dystrophies; hence, keratoplasty is performed at a younger age and seems to have a lower recurrence rate than lattice and granular dystrophies [49]. Furthermore, secondary to the early manifestation of the disease and the high recurrence rate of PTK, many patients will end up with keratoplasty, hence a higher number of published studies on the outcome of keratoplasty in MCD. Apart from sporadic case reports, several large-scale case series have assessed the short-term and long-term outcomes of penetrating and lamellar keratoplasty in MCD.

Penetrating keratoplasty may survive up to 50 years, according to Bischoff-Jung et al. [106]. They reported a case of a 76-year-old male who presented 50 and 46 years after receiving a small 6 mm PK in his right and left eye, respectively. This is the most prolonged reported survival for keratoplasty in macular dystrophy. Klintworth et al. reported recurrence of macular dystrophy 18 and 19 years after PK and DALK in two patients who received the transplant in their early twenties [107]. Recurrences may occur many years after PK due to the migration of keratocytes carrying the gene defect from the host to the donor cornea [107]. Therefore, the graft’s periphery is the first and most severely affected part in the recurrence of the disease after PK. Al-Swailem et al., in a large-scale study, assessed the outcome of PK in 229 eyes of patients with macular dystrophy who were followed up for 1–17 years [108]. They reported a very good prognosis of PK in this type of dystrophy, as clinically significant recurrence was observed in only 12 (5.2%) grafts after a mean interval of 84 ± 48.2 months. They noticed a significantly higher risk of recurrence in patients older than 40 years at the time of surgery and those with smaller grafts, i.e., ≤7.0 mm.

Another large-scale case series of PK for corneal dystrophies, including 53 cases of MCD and four of GCD that were followed up for an average of 28 months, was published by Pandrowala et al. [98]. No disease recurrence was noticed in that short-term follow-up duration, and graft survival was reported at 92% and 75% in MCD and GCD, respectively.

Akova et al. published the long-term outcome of 5–8 mm PK in 31 patients with macular dystrophy [109]. The mean age at the time of the first keratoplasty in their series was 41 ± 4 years, and the mean follow-up period was 149 ± 29 months. They noticed an approximately 20% recurrence rate at the mean period of 182 months, which, similarly to the above study, was more common in smaller grafts. This is because smaller grafts are more readily invaded by abnormal glycoproteins and/or abnormal stromal cells. They revealed that there is a 50% chance of recurrence within a graft at 18 years.

With the evolution of lamellar keratoplasty, DALK was tried in macular dystrophy to overcome some of the disadvantages of PK, such as loss of ocular integrity, increased intraocular pressure, cataract and higher risk of endothelial cell loss and immunologic rejection [110]. Robin’s work is probably one of the oldest reports of lamellar keratoplasty in MCD in both eyes of a young patient that showed a significant recurrence 11 years later and had to be replaced with a PK. With 0.5 mm thickness, it provided a good outcome for 11 years [111], even though MCD recurs earlier after lamellar keratoplasty (LKP) than after PK. Following keratoplasty, the recurrence of the disease depends on some factors such as residual genetically defective keratocytes in the host cornea (which can migrate to the graft), the size of the graft, and the length of the follow-up. While in PK, the recurrence starts from the graft margins, in lamellar keratoplasty, the posterior graft–host interface is another source of recurrence besides the graft’s peripheral edge. It was demonstrated that whereas the recurrence in PK starts with opacities at the graft–host junction with gradual extension towards the centre of the cornea, the recurrence in DALK starts at the graft–junction interface with further anterior extension [110]. Therefore, it is suggested that the decision between PK and DALK should be made based on the severity of the disease and the extent of posterior stromal involvement, which can be demonstrated on ASOCT or UBM [101]. However, as DALK is a technically more challenging surgery with a steep learning curve, the surgeon’s experience and preference also have a role in defining the management plan. There are some sporadic case reports of DALK in MCD [106,111,112]. In 2006, a Japanese case series compared 14 PK and 10 DALK in MCD with a follow-up duration of 29.5 and 55.4 months in age-matched groups and 7.75 mm grafts. There were no detailed data on disease recurrence in each group, but a generally higher survival was reported in the PK group compared to the DALK group [47]. Additionally, Kodavoor et al. reported a good visual outcome of DALK in 14 eyes with MCD 1-year post-op, with no recurrence [113]. This is a very short follow-up, and nonetheless, no recurrence is expected 1 year after keratoplasty.

Unal et al. published a case series of 69 eyes with 43 macular, 9 granular and 17 lattice dystrophies that underwent DALK to restore vision [48]. Big bubble technique was applied in all cases; this was accomplished in 81% of eyes, while the rest of the eyes had a manual dissection. The graft size varied between 7 and 8.5 mm. All eyes had a better vision at 6 months post-op. The recurrence of the disease occurred in one eye with macular dystrophy (2.3%), six eyes with lattice dystrophy (35.3%), and two eyes with granular dystrophy (22%) within 43.5 ± 23.9 months.

In the only randomized clinical trial on keratoplasty in MCD, Sogutlu et al. compared the visual outcome of 35 DALK and 41 PK in MCD [114]. Only patients without endothelial involvement were enrolled in the study. The patients were followed up for 30.5 ± 8.75 and 31.2 ± 9.78 months in the DALK and PK groups, respectively. They reported comparable BCVA and contrast sensitivity between groups but higher levels of higher-order aberrations with DALK and a higher rate of endothelial cell loss with PK. They reported 8.5% and 12% graft rejection in DALK and PK groups and 2.8% and 7.3% re-graft rates, respectively, secondary to graft failure. The authors noticed a fairly similar recurrence rate of the disease at 5.7% and 4.8% with DALK and PK, respectively. It is interesting to note that in a controlled study with the exclusion of eyes with endothelial involvement, the recurrence rate is not significantly different between groups in mid-term follow-up.

A long-term interventional case series in 2013 compared the outcomes of 57 PK with 21 DALK (using lamellar dissection) in patients with MCD [110]. Since DALK was introduced after PK, there were fewer patients in that group with shorter follow-up (mean follow-up of 5.7 ± 4.5 after PK and 3.4 ± 2.1 years after DALK). The authors noticed a significantly better BCVA and less recurrence rate in PK cases (7.7% in 5 years compared to 49.5% in the DALK group) but a better endothelial cell count and fewer post-op complications with DALK. This is the highest rate of recurrence of MCD reported following DALK, and the authors believe including both eyes of each patient to increase the sample size may have had a role in the high recurrence rate. They also noticed significantly less recurrence with larger grafts (8.4% in 7 mm or smaller, compared to 1.9% in 7.25 mm or larger grafts). Unlike a previous study [108], they noticed a higher recurrence rate in younger patients than in older ones. Considering their very high rate of disease recurrence with DALK, they suggested a PK for severe disease in young patients and a DALK for less severe disease in older patients.

Likewise, the authors of another retrospective case series reported the outcomes of their 109 PK and 21 DALK cases for MCD with a follow-up of 43 ± 24 months versus 17 ± 8 months, respectively [115]. As widely recognised with PK and DALK, intra-operative and immediate post-op complications were higher in the DALK group, but post-op complications such as endothelial rejection, secondary glaucoma and endophthalmitis were significantly higher in the PK group. Clearly, all of these complications increase the likelihood of graft failure over time. At 4 years, the graft survival was 78% versus 70% for PK and DALK, respectively. The survival rate for DALK in this study was lower than the reported average, which was attributed to the higher rates of the postoperative double anterior chamber (43%) as compared with those reported in the literature secondary to a steep learning curve of DALK.

Recently, another large-scale case series compared the outcome of 22 DALK with 135 PK, in which the patients were followed up for 7.2 ± 6.2 years and 9.7 ± 4.1 years, respectively [116]. Similarly to the above studies, there was an unequal distribution of cases between the groups, with shorter follow-ups for DALK eyes. However, both groups had comparable visual acuity, significant recurrence rate (2.9% for PK eyes and 4.5% for DALK eyes) and graft survival (95.5% for PK and 91.1% for DALK) at the last visit.

In summary, PTK provides a rapid and excellent visual recovery in patients with MCD; however, this excellent visual outcome is spoiled by the very high rate of recurrence within a short time scale. This treatment modality may be used and repeated to delay a graft in virgin eyes or postpone a re-graft in transplanted corneas. Keratoplasty is a long-term management plan to restore vision in MCD, with a reasonably good outcome based on the published data. Theoretically, PK should have a better prognosis with less recurrence than DALK, as the only source of recurrence is the graft–host junction, while the graft–host interface is an extra source of recurrence with DALK. However, the only prospective study in this field showed that good patient selection, i.e., exclusion of eyes with endothelial involvement, can reduce the recurrence rate in DALK to the PK level and yield a better graft survival due to less rejection and endothelial failure. Although more prospective studies with a greater number of DALK cases are required to assess the validity of this point, one may consider PK in the presence of endothelial involvement and spare DALK for eyes where the endothelium is intact. Although there is no agreement on graft prognosis based on the age of the patients, the majority of case series confirm that a larger graft carries less risk of recurrence.

Tables S6 and S7 summarise published papers on the outcome of PTK and keratoplasty in MCD.

## 8. Schnyder Corneal Dystrophy

Schnyder corneal dystrophy (SCD) is a rare, autosomal dominant, anterior stromal dystrophy caused by a mutation in the *UBIAD1* gene on chromosome 1*p*. It is characterized by the accumulation of crystals in corneal stroma containing cholesterol and neutral fat, which stains red with oil red O. Clinically, the crystals appear yellow and white and can be associated with corneal haze and corneal arcus. SCD presents in childhood with slow progression over years, causing reduced visual acuity and glare. The condition may be associated with systemic hypercholesterolaemia, which has been reported in 66% of patients [117,118]. However, interestingly, the extent of corneal opacification is not associated with a likelihood of concurrent dyslipoproteinemia [119].

According to electron microscopy studies, Schnyder corneal dystrophy is characterized by disruption of the superficial corneal stroma and Bowman’s zone, whereas the epithelium, endothelium, and Descemet’s membrane are unaffected [120], which would suggest that anterior lamellar keratoplasty would be the treatment of choice. Nonetheless, interestingly, Freddo and colleagues (1989) described focal areas of endothelial cell degeneration in some cases of SCD producing small discontinuities in the endothelial cell layer of Descemet’s membrane [121].

Establishing whether there is co-existing hyperlipidaemia is important not only to reduce patients’ cardiovascular risk factors but also to choose the appropriate ophthalmic treatment. A high serum level of cholesterol can be a contraindication to keratoplasty, and cholesterol has been shown to accumulate much more rapidly in the abnormal cornea of cholesterol-fed rabbits [122] as well as in corneal grafts of patients with hyperlipidaemia [123].

### 8.1. PTK

Paparo and colleagues reported successfully treating four eyes of three patients with Schnyder’s dystrophy with PTK [124]. Visual improvement from average Snellen best corrected visual acuity of 20/175 to 20/40 was noted post-op with significant reduction of glare and photophobia in all patients. No recurrence was reported in the study follow-up period, which ranged from 7 months to 3 years. The authors reported a post-operative hyperopic shift of approximately +3 dioptres and one case of irregular astigmatism, which was due to eccentric ablation [124].

In Weiss’s case series, five eyes of three patients with Schnyder’s dystrophy were treated with PTK. Preoperative BCVA ranged from Snellen 20/50 to 20/60 in the four eyes where Schnyder’s dystrophy was the only ocular pathology. One patient required PK at 1 year due to persistent corneal haze post-PTK [125].

Köksal et al. (2004) reported a good visual outcome after treating two eyes of one patient with Schnyder’s dystrophy with PTK [126].

Recurrence of Schnyder’s dystrophy post-PTK is very infrequent, with no reports of recurrence at 6 months [9], 3 years [124] or 5 years [126].

### 8.2. Keratoplasty

There is scarce literature on the outcome of penetrating keratoplasty and no data on the outcome of DALK in SCD. Kitazawa et al. reported long-term outcomes following penetrating keratoplasty in six eyes of five patients with Schnyder’s dystrophy confirmed by genetic analysis [127]. The authors reported BCVA of 0.02 LogMar in all eyes at 3 years, which was a significant improvement from preoperative visual acuity (1.7 to 0.22 LogMar). There was no disease recurrence or graft rejection between 3 and 10 years of follow-up in their cohort.

Similarly, Weiss conducted a retrospective analysis of 115 patients with Schnyder’s dystrophy and described the post-op outcomes in 20 patients who were treated with penetrating keratoplasty [125]. Most of them did not require treatment until later in life, in the 7th decade, with the main indication being glare and reduced photopic vision. Good post-op visual acuity was achieved, with 72% of patients achieving VA of 20/50 or above. Recurrence occurred in 21% of patients within the mean follow-up period of 5 years, ranging from 1 to 8 years.

Conversely, no recurrence in graft was reported by Marcon et al. after 4.5 years [128]; however, their study only included one patient with Schnyder’s dystrophy.

In summary, the body of evidence for both treatment modalities of Schnyder’s dystrophy—keratoplasty and PTK—is small and only comprises retrospective case reports and case series with no prospective comparative studies or randomised controlled trials. Considering the very good visual outcome and low recurrence rate with PTK, and to avoid the hassles of keratoplasty, PTK should be tried first in restoring the vision in SCD. In case of frequent recurrence, one can offer keratoplasty, bearing in mind the risk of recurrence in the graft.

Table S8 summarises published papers on the outcome of PTK and keratoplasty in SCD.

## 9. Conclusions

In epithelial-stromal and stromal corneal dystrophies, where there is visual reduction, treatment options may include either PTK or corneal transplantation. Although recurrence of the disease can occur with both treatment modalities, PTK is the treatment of choice for anterior dystrophies, has the advantage of being less invasive and can be repeated in cases of recurrence. On the other hand, keratoplasty provides a longer visual rehabilitation before the recurrence of the dystrophy, where deeper layers of the stroma are involved.

- Due to the anterior location of the deposits in Reis–Bücklers and Thiel–Behnke dystrophies, good visual outcomes can be achieved with PTK.
- For lattice, Avellino, granular and macular corneal dystrophies, PTK laser may provide temporary visual improvement; however, with recurrences, there may be the need for further PTK or a corneal transplant. Between PK and DALK, treatment choice is often debatable. PK has the advantage of being technically less challenging surgery with complete removal of the host stroma but a higher rejection risk and hence a lower graft survival. Contrarily, DALK carries a lower risk of graft rejection and hence a better survival, while it is a technically more challenging procedure and carries an additional risk of recurrence through the graft–host interface secondary to the residual genetically defective cells. Meticulous patient selection with pre-op ASOCT to detect the posterior extent of the dystrophy and using Descemet-baring dissection techniques may decrease the recurrence rate in DALK up to the PK level.
- PTK offers good visual outcomes and allows one to delay the keratoplasty in LCD. Although the recurrence rate is high, the time to recurrence is relatively slow, allowing several re-treatments. Therefore, it is recommended to start with PTK and re-treatment with PTK before proceeding to keratoplasty when possible. FLK appears to be a beneficial option that also allows for the correction of irregular astigmatism simultaneously. When moving to keratoplasty, DALK is preferred to PK for better graft survival; however, the recurrence rate is not fully understood yet.
- In GCD, to remove the lesions that are confined to the anterior stroma, PTK may be offered and repeated after recurrence. FLK is an alternative to PTK in such cases. ALK and DALK are good choices where the stromal involvement is not too deep, and PK may be offered to restore the vision in deep stromal or pre-Descemet involvement. GCD has been shown to recur both from subepithelial and graft–host interface origins. Limbo-keratoplasty is another suggested treatment modality to prevent epithelial recurrence in GCD.
- The recurrence rate with PTK in Avellino dystrophy is high, and big bubble DALK may be the treatment of choice. Corneal electrolysis is another described procedure in the treatment of Avellino dystrophy.
- In MCD, PK may be the preferred treatment because of the likelihood of endothelial involvement.
- For Schnyder dystrophy, should treatment be required, PTK may be the preferred option due to the potential for recurrence of the disease in corneal transplantation.

## Supplementary Materials

The following supporting information can be downloaded at: https://www.mdpi.com/article/10.3390/vision7010022/s1, Figure S1: Anterior segment photo of RBCD (left) and OCT (right) showing irregular hyperreflective material at the Bowman layer; Figure S2: Anterior segment photo of GCD of the right eye with old penetrating keratoplasty (top left) and left virgin cornea (top right), ASOCT of the right eye with recurrence of the disease and endothelial involvement (bottom left) and left eye with granular hyperreflective deposits in the stroma (bottom right); Table S1: Summary of published papers on the outcome of PTK and keratoplasty in RBCD; Table S2: Summary of published papers on the outcome of PTK and keratoplasty in TBCD; Table S3: Summary of published papers on the outcome of PTK and keratoplasty in LCD; Table S4: Summary of published papers on the outcome of PTK in GCD; Table S5: Summary of published papers on the outcome of keratoplasty in GCD; Table S6: Summary of published papers on the outcome of PTK in MCD; Table S7: Summary of published papers on the outcome of keratoplasty in MCD; Table S8: Summary of published papers on the outcome of PTK and keratoplasty in SCD.
