# Supplementary material for: Management of Stromal Corneal Dystrophies; Review of the Literature with a Focus on Phototherapeutic Keratectomy and Keratoplasty
Source: Vision (Basel). 2023 Mar 13;7(1):22. doi: 10.3390/vision7010022 (PMC10059954; doi:10.3390/vision7010022)
Supplement: Supplementary file 1 [file vision-07-00022-s001.zip › vision-2063035-supplementary Figures.pdf]

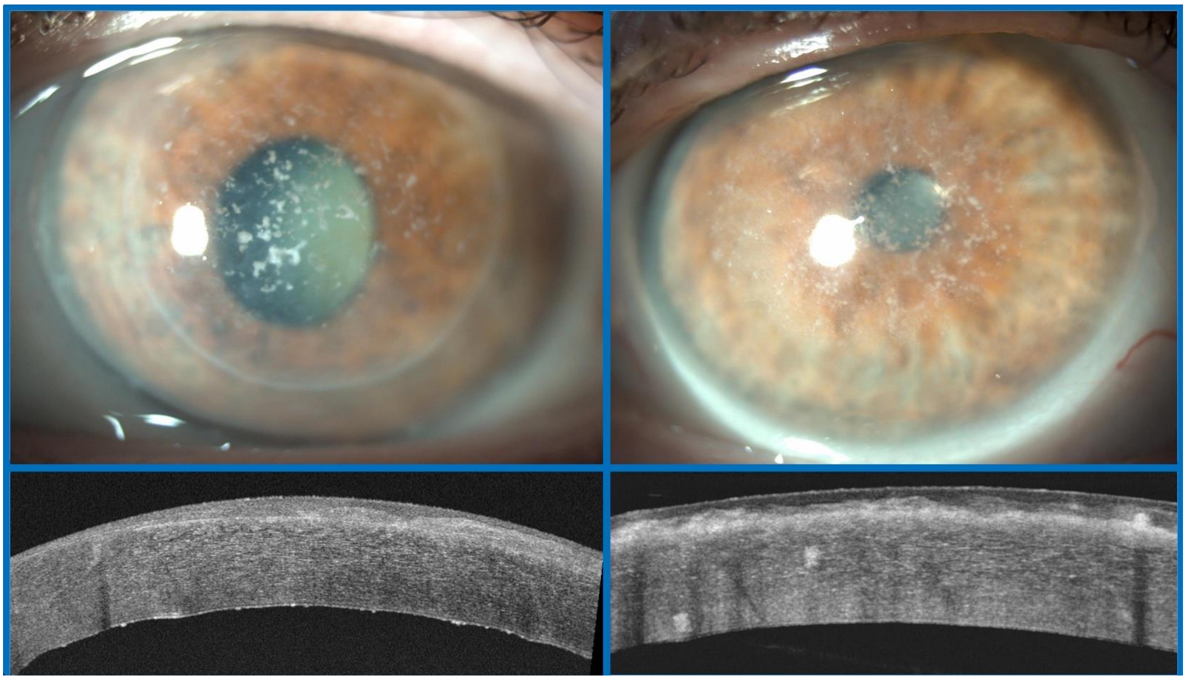

Figure S1. Anterior segment photo of RBCD (left) and OCT (right) showing irregular hyperreflective material at the Bowman layer.

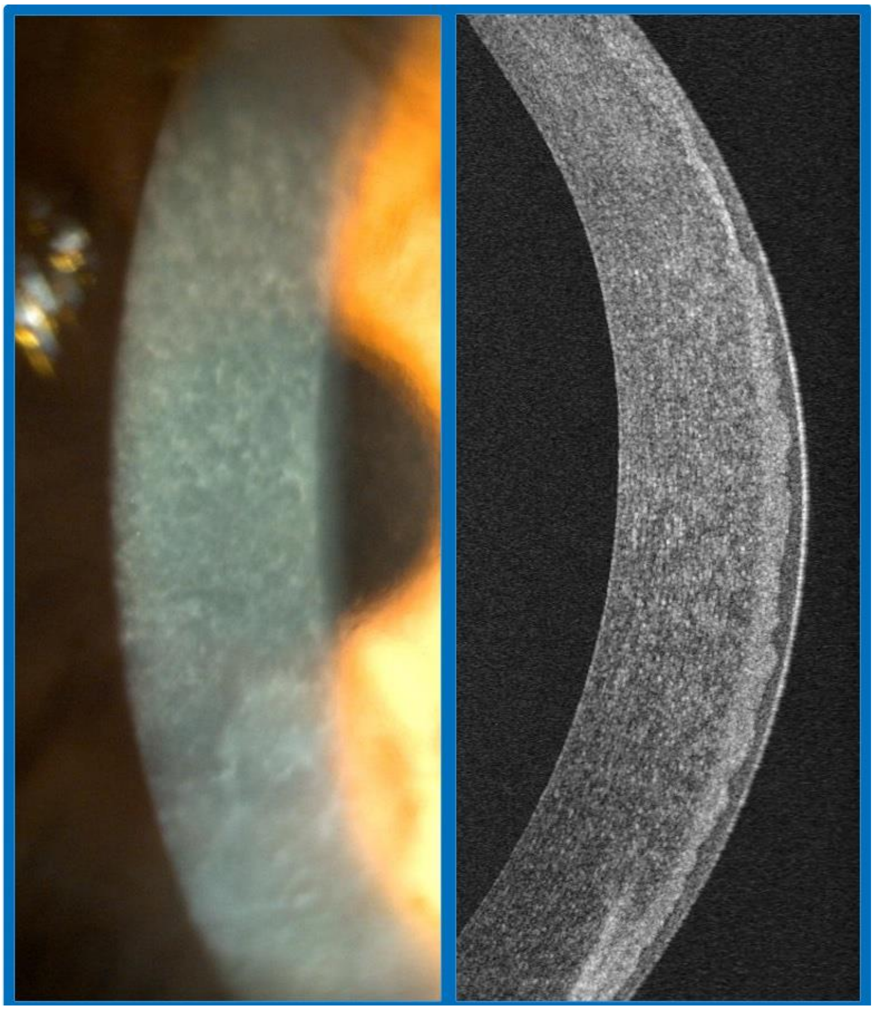

Figure S2. Anterior segment photo of GCD of the right eye with old penetrating keratoplasty (top left) and left virgin cornea (top right), ASOCT of the right eye with recurrence of the disease and endothelial involvement (bottom left) and left eye with granular hyperreflective deposits in the stroma (bottom right).
